# Berberine regulates UCP1 expression by reshaping the conformation of quadruplex formed by the element of the *UCP1* gene promoter: Berberine, a novel regulator of UCP-1

**DOI:** 10.3724/abbs.2022155

**Published:** 2022-11-02

**Authors:** Qiuhui Wang, Xingke Xu, Xiaoqing Jiang, Fei Yin

**Affiliations:** 1 Chongqing Key Laboratory of Medicinal Chemistry & Molecular Pharmacology Chongqing University of Technology Chongqing 400054 China; 2 College of Pharmacy and Bioengineering Chongqing University of Technology Chongqing 400054 China

Obesity has become a serious issue due to its worldwide prevalence and is the leading risk factor for other metabolic diseases. Increasing evidence indicates that the activation and recruitment of brown adipose tissue (BAT) and the induction of the expression of uncoupling protein 1 (UCP1) are attractive strategies to increase metabolic efficiency and counteract weight gain [1]. UCP1, a thermogenic protein, is located in the inner mitochondrial membrane, is mainly expressed in brown and beige/brite adipose tissues and plays critical roles in metabolic and energy balance by releasing chemical energy as heat [2]. Furthermore, the activity of the *UCP1* promoter has been shown to be activated during the browning of white adipose tissue (WAT) and is generally inactivated in tissues except brown and beige/brite adipose tissues [3]. Therefore, targeting the activity of the *UCP1* gene promoter might be a promising strategy to regulate the expression of UCP1.

G-quadruplexes are nucleic acid secondary structures formed in G-rich sequences in DNA or RNA, which include single nucleic acid strands (unimolecular G-quadruplex, Uni-G4), between strands (bimolecular G-quadruplex, Bi-G4), and tetramolecular quadruplexes (tetra-G4). G-quadruplexes have become promising therapeutic targets due to their roles in the expressions of many critical genes. There is a G-quadruplex-forming sequence in the promoter of the *UCP1* gene, and mutation in the G-quadruplex sequence dramatically enhances the activity of the *UCP1* gene promoter, suggesting that the G-quadruplex is a potential target to regulate UCP1 expression [4].

Berberine has been shown to induce the development of beige/brite adipocytes by increasing the expression of UCP1 and proved to have metabolic benefits by increasing energy expenditure and limiting weight gain in obese and/or db/db mice [ 5, 6] . Although the roles of transcription cascades and epigenetics in the regulation of the activity of the *UCP1* gene promoter have been widely reported, it is not clear whether berberine affects the activity of the *UCP1* gene promoter and its associated mechanisms.

In this study, we characterized the effects of berberine on the G-quadruplex formed by the oligonucleotide fragment of the *UCP1* gene promoter (Olig) using a variety of spectroscopic techniques (UV‒visible, fluorescence, and circular dichroism) and native gel electrophoresis approaches. We also determined the influence of berberine on the expression of the *UPC1* gene and the biosynthesis of fatty acids in differentiated 3T3-L1 adipocytes.

The oligonucleotide fragment of the *UCP1* gene promoter (CGAGGGTGGGTAGGAGGGGACGCGGGGACT, Olig) and its G→A mutated sequence (CGAGGGTAAATAGGAAAAAACGCAAAAACT, muOlig) were synthesized by Sangon Biotech (Shanghai, China), dissolved in double-distilled water, and prepared in 10 mM Tris-HCl buffer (pH 7.4). After the DNA samples were heated to 95°C for 5 min, the indicated dosages of berberine and/or KCl were mixed with the DNA samples before the DNA was slowly cooled to room temperature. Absorption spectroscopy was recorded using a Shimazu 2450 UV‒vis spectrophotometer at 25°C from 280 to 550 nm. As shown in Figure 1A, there were two distinct peaks of berberine solution at 340 and 420 nm; the addition of quadruplex caused a significant hypochromicity (approximately 45%) and a moderate bathochromic shift of 8 nm for the high-energy peak from 340 to 348 nm and hyperchromicity with a redshift of 50 nm for the low-energy peak from 420 to 470 nm. These data suggest that berberine binding with oligonucleotide fragments results in hypochromicity and bathochromism through a strong intercalation between berberine and the G-quadruplex. At the same time, fluorescence spectroscopy was performed on a fluorescence spectrometer (Thermo Fisher Scientific, Waltham, USA) from 450 to 700 nm at an excitation wavelength of 280 nm at 25°C, and the excitation and emission slit widths were set as 5 and 10 nm, respectively. As shown in Figure 1B, berberine alone in Tris buffer was nonfluorescent, but in the presence of G-quadruplexes, the fluorescence intensity of berberine was increased dramatically, and the λ _max_ in the fluorescence emission spectra shifted to the blue end by 8 nm. Moreover, the effect of berberine on the conformation of the G-quadruplex formed by Olig was further evidenced by CD spectroscopy, which was recorded on a spectropolarimeter (CHIRASCAN; Applied Photphysics Ltd, Leatherhead, UK) from 200 to 350 nm at 25°C in a 0.1 cm path length quartz cell at a scanning rate of 20 nm/min. As shown in Figure 1C, CD spectroscopy exhibited a characteristic peak of the G-quadruplex at 260 nm in the presence of potassium, a known G-quadruplex stabilizer [7], while berberine partly increased the intensity of the peak at 260 nm.

**Figure 1:**
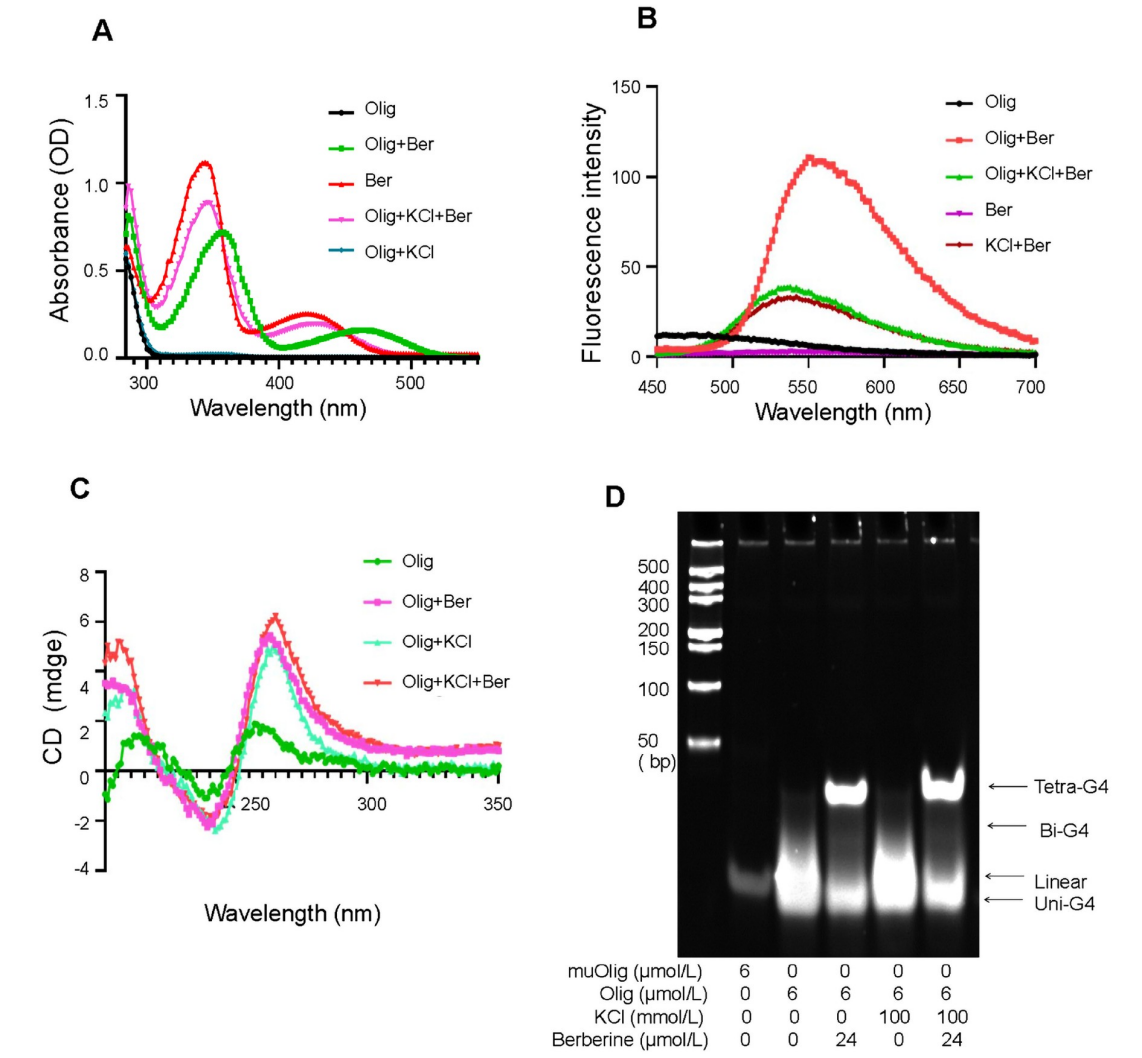
Figure 1 Characterization of quadruplexes formed by the oligonucleotide fragment in the presence or absence of berberine After 6 μM oligonucleotide fragment of the UCP1 gene promoter (Oligo) dissolved in 10 mM Tris-HCl buffer (pH 7.4) was mixed with 100 mM KCl in the presence or absence of 24 μM berberine (Ber) and incubated overnight at 4°C, the UV‒visible (A), fluorescence (B) and CD (C) were rescored, respectively, and native gel electrophoresis was performed to distinguish the conformation of quadruplexes (D).

G-quadruplexes are compact structures, so they migrate faster than non-G-rich oligonucleotides with the same length [8]. To further distinguish the effects of berberine on the conformation of the G-quadruplex formed by Olig, we used native gel electrophoresis to observe the G-quadruplex structure with or without berberine. The results indicated that in the presence of 100 mM KCl, almost all the G-quadruplexes formed by Olig were Uni-G4, but berberine caused a transformation of G-quadruplexes from Uni-G4 to Tetra-G4 ( Figure 1D).

Mouse-derived 3T3-L1 preadipocytes, purchased from the China Center for Type Culture Collection (Wuhan, China), were induced to differentiate into adipocyte-like cells before being used by incubating with 0.5 mM isobutyl methylxanthine (IBMX, ab120840; Sigma, St Louis, USA) and 1 μM dexamethasone (Sigma) for 3 days. Then, the medium was replaced with complete media plus 10 μg/mL insulin (MCE, Monmouth Junction, USA), and the cells were cultured for another 3 days. Before determining the effect of berberine on the expression of UCP1, we initially evaluated the cytotoxicity of berberine in differentiated 3T3-L1 adipocytes by the 3-(4,5-dimethylthiazol-2-yl)-2,5-diphenyltetrazolium bromide (MTT) colorimetric assay after the cells were cultured with the indicated doses of berberine for 24 h. The results demonstrated that, once the concentration of berberine was over 20 μM, berberine showed noticeable cytotoxicity (data not shown). Then, we determined the effect of berberine on the activity of the *UCP1* gene promoter with a reporter gene assay. Generally, after the promoter of the human *UCP1* gene was cloned and transfected into HEK293 cells using Lipofectamine 3000 (Invitrogen, Carlsbad, USA) and incubated for 48 h, the luciferase activity was determined with the Dual-Glo® Luciferase Assay System (Promega, Madison, USA) according to the supplier’s suggestion. The data revealed that berberine treatment evidently increased the activity of the *UCP1* gene promoter ( Figure 2A). Furthermore, incubation with berberine increased the expression of UCP1 in a time- and dose-dependent manner ( Figure 2B‒D), and treatment with 10 μM berberine for 6 h increased the mRNA level of *UCP1* by approximately 4.3-fold ( Figure 2B). Incubation with 10 μM berberine for 72 h increased the protein level of UCP1 by approximately 2–2.5 folds ( Figure 2C,D).

**Figure 2:**
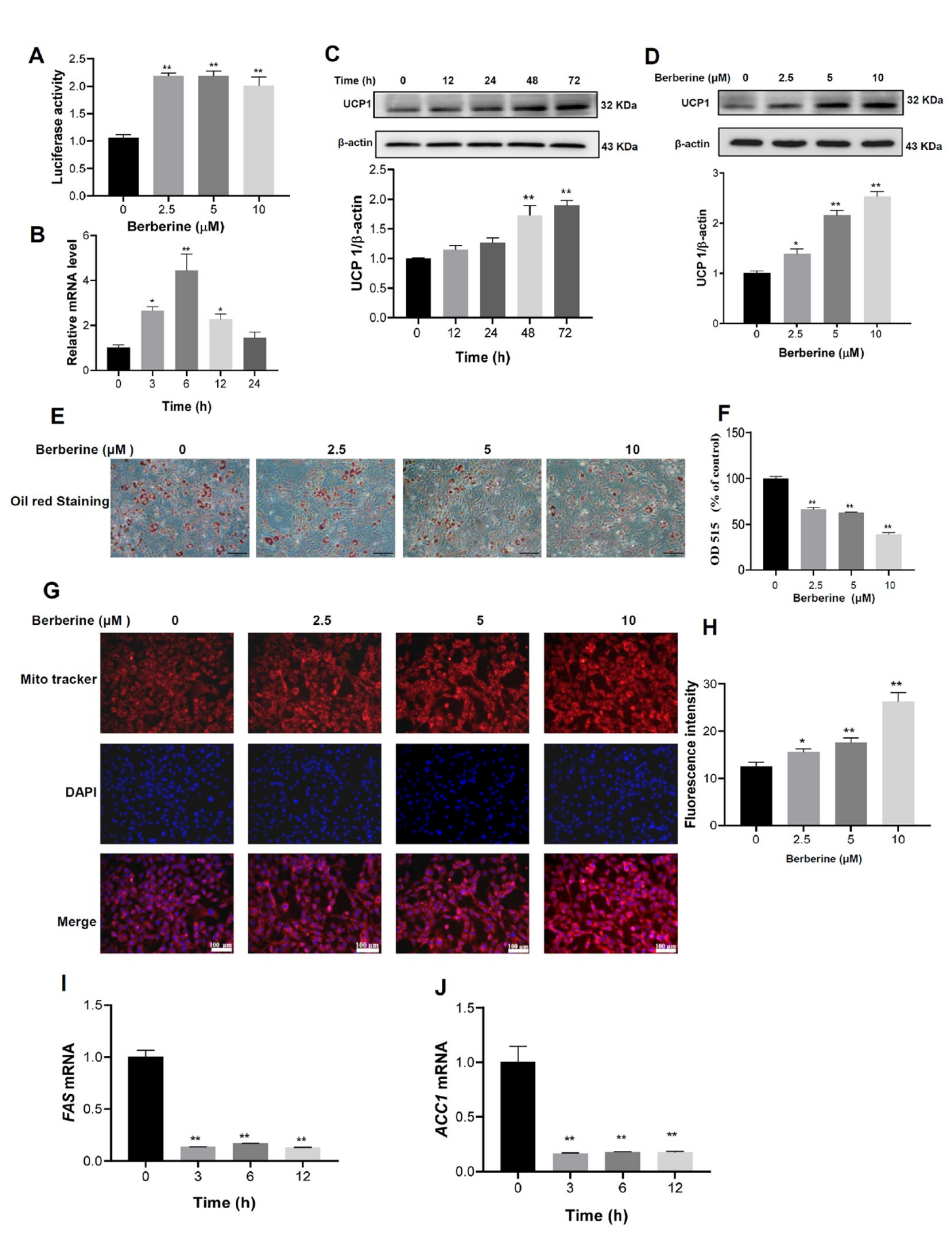
Figure 2 Berberine increased the expression of UCP1 to inhibit fatty acid biosynthesis in differentiated 3T3-L1 adipocytes (A) After the UCP1 gene promoter was cloned and transfected into HEK293 cells, the promoter activity of the UCP1 gene was determined by Dual-Glo Luciferase Assay according to the supplier’s suggestions. (B‒D) After differentiated 3T3-L1 adipocytes were incubated with the indicated dose of berberine (10 μM for C and D) for 0, 12, 24, 48 or 72 h, the mRNA and protein levels of UCP1 were determined by qRT-PCR and western blot analysis, respectively. (E‒H) After differentiated 3T3-L1 adipocytes were treated with berberine for 48 h, oil red O staining was used to detect the effect of berberine on the accumulation of lipid droplets. Scale bar= 100 μm (E), and after the cells were incubated with isopropanol, the absorbance was determined at 515 nm with a microplate reader (F). The mitochondrial content was observed by MitoTracker staining and DAPI was used to stain the cell nuclei Scale bar= 100 μm (G), and the fluorescence intensity was measured with a microplate reader (H). (I,J) After differentiated 3T3-L1 adipocytes were incubated with 10 μM berberine for 0, 3, 6 and 12 h, the mRNA levels of FAS (I) and ACC1 (J) genes were determined by qRT-PCR. Data are shown as the mean±SD (n=4). * P<0.05, and ** P<0.01 vs control.

Accumulating evidence indicates that the browning/beigeing of WAT can be leveraged to treat obesity due to its distinctive capacity to drive thermogenesis, and UCP1 is the best thermogenic effector in adipocytes, which uncouples respiration from ATP synthesis, dissipates the proton gradient as heat to maintain body temperature in a cold environment or wastes energy [9]. Meanwhile, BAT is rich in mitochondria and plays a potential role in energy expenditure and glucose homeostasis, and UCP1 has been used as a hallmark protein in the browning/beigeing of WAT [10].

To explore the actions of berberine that regulate the expression of UCP1 in adipocytes, differentiated 3T3-L1 adipocytes were cultured with 0, 2.5, 5 and 10 μM berberine for 48 h. After that, the cells were fixed with fresh 4% paraformaldehyde for 10 min and then stained with oil red O for 20 min. The results showed that treatment with berberine for 48 h decreased the accumulation of lipid droplets in a dose-dependent manner in differentiated 3T3-L1 adipocytes ( Figure 2E,F). Interestingly, incubation with berberine also dose-dependently increased the content of mitochondria in differentiated 3T3-L1 adipocytes ( Figure 2G,H).

To clarify the mechanisms by which berberine attenuates the accumulation of lipid droplets in differentiated 3T3-L1 adipocytes, the expression of fatty acid synthesis ( *FAS*) and acetyl-CoA carboxylase 1 ( *ACC1*), the two key genes for the biosynthesis of fatty acids, were determined by qRT-PCR. Generally, the cells were incubated with 10 μM berberine for the indicated time, and total RNA was extracted using TRIzol reagent (Invitrogen). First-strand cDNA was generated using the random hexamer primer provided with an iScript cDNA synthesis kit (Bio-Rad, Hercules, USA). The qPCR experiments were conducted on a Step-One plus real-time PCR system and carried out using a final volume of 10 μL, containing 1 ng of reverse-transcribed total cDNA, 2 nM of forward and reverse primers, and SYBR green PCR master mixture, which was performed in 96-well plates using the CFX96 real-time PCR system (Bio-Rad). The PCR conditions consisted of 40 cycles with 15 s denaturation at 95°C, 30 s annealing at 55°C, and 60 s extensions at 72°C. The fold change in mRNA was calculated by the 2 ^–∆∆Ct^ method using β-actin as the reference gene to normalize the data for all samples. The specific primers were *mACC1* (forward: 5′-GAATCTCCTGGTGACAATGCTTATT-3′, reverse: 5′-GGTCTTGCTGAGTTGGGTTAGCT-3′), *mFAS* (forward: 5′-TGGGTTCTAGCCAGCAGAGT, reverse: TACCACCAGAGACCGT TATGC-3′), and *β-actin* (forward: 5′-GGCTGTATTCCCCTCCATCG-3′, reverse: 5′-CCAGTTGGTAACAATGCCATGT-3′). Our data indicated that treatment with berberine significantly decreased the mRNA levels of *FAS* and *ACC1* genes in differentiated 3T3-L1 adipocytes ( Figure 2I,J).

In summary, the spectroscopic characteristics demonstrated that in the presence of Olig, the solution of berberine showed a significant hypochromicity and a moderate bathochromic shift for the high-energy peak at 340 nm and hyperchromicity and a redshift of the low-energy peak at 420 nm. The fluorescence intensity of the berberine solution was also dramatically increased by the addition of quadruplexes formed by Olig. All of these spectroscopic data suggested that berberine might not be a good quadruplex DNA intercalator but rather a groove binder. Meanwhile, native gel electrophoresis indicated that berberine caused a significant transformation of G-quadruplexes from Uni-G4 to Tetra-G4. Furthermore, together with increasing the content of mitochondria, berberine treatment evidently increased the activity of the *UCP1* gene promoter, induced the expression of UCP1 in a dose- and time-dependent manner, and attenuated the accumulation of lipid droplets by reducing the expression of FAS and ACC1 in differentiated 3T3-L1 adipocytes. This study suggested that the metabolic benefits of berberine, by increasing energy expenditure and limiting weight gain in obese and/or db/db mice, could be involved in its role in the transformation of quadruplexes formed by the *UCP1* gene promoter and its expression.

## References

[1] Brandao CFC, Carvalho FG, Souza AO, Junqueira‐Franco MVM, Batitucci G, Couto‐Lima CA, Fett CA (2019). Physical training,
*UCP1* expression, mitochondrial density, and coupling in adipose tissue from women with obesity. Scand J Med Sci Sports.

[2] Gonzalez-Barroso MDM, Ricquier D, Cassard-Doulcier AM (2000). The human uncoupling protein-1 gene (UCP1): present status and perspectives in obesity research. Obesity Rev.

[3] Brondani LA, Assmann TS, Duarte GCK, Gross JL, Canani LH, Crispim D (2012). The role of the uncoupling protein 1 (UCP1) on the development of obesity and type 2 diabetes mellitus. Arq Bras Endocrinol Metab.

[4] Zhao Y, Uhler JP (2018). Identification of a G-quadruplex forming sequence in the promoter of UCP1. Acta Biochim Biophys Sin.

[5] Ilyas Z, Perna S, Al-thawadi S, Alalwan TA, Riva A, Petrangolini G, Gasparri C (2020). The effect of Berberine on weight loss in order to prevent obesity: A systematic review. Biomed Pharmacother.

[6] Zhang Z, Zhang H, Li B, Meng X, Wang J, Zhang Y, Yao S (2014). Berberine activates thermogenesis in white and brown adipose tissue. Nat Commun.

[7] Marathias VM, Bolton PH (2000). Structures of the potassium-saturated, 2:1, and intermediate, 1:1, forms of a quadruplex DNA. Nucleic Acids Res.

[8] Burge S, Parkinson GN, Hazel P, Todd AK, Neidle S (2006). Quadruplex DNA: sequence, topology and structure. Nucleic Acids Res.

[9] Chouchani ET, Kazak L, Spiegelman BM (2019). New advances in adaptive thermogenesis: UCP1 and beyond. Cell Metab.

[10] Wang GX, Meyer JG, Cai W, Softic S, Li ME, Verdin E, Newgard C (2019). Regulation of UCP1 and mitochondrial metabolism in brown adipose tissue by reversible succinylation. Mol Cell.

